# Causal Association Between Body Mass Index and Kidney Stone Disease in Taiwanese: A Mendelian Randomization Study

**DOI:** 10.7150/ijms.128104

**Published:** 2026-03-17

**Authors:** Ming-Ru Lee, Po-Yu Hsu, Szu-Chia Chen, Shu-Pin Huang, Jiun-Hung Geng

**Affiliations:** 1 Department of Urology, Kaohsiung Municipal Siaogang Hospital, Kaohsiung 812015, Taiwan.; 2 Department of Urology, Kaohsiung Medical University Hospital, Kaohsiung Medical University, Kaohsiung 807378, Taiwan.; 3 Department of Post-Baccalaureate Medicine, College of Medicine, Kaohsiung Medical University, Kaohsiung 807378, Taiwan.; 4 Department of Internal Medicine, Kaohsiung Municipal Siaogang Hospital, Kaohsiung Medical University, Kaohsiung 812015, Taiwan.; 5 Department of Internal Medicine, Division of Nephrology, Kaohsiung Medical University Hospital, Kaohsiung Medical University, Kaohsiung 807378, Taiwan.; 6 Faculty of Medicine, College of Medicine, Kaohsiung Medical University, Kaohsiung 807378, Taiwan.; 7 Center for Big Data Research, Kaohsiung Medical University, Kaohsiung 807378, Taiwan.; 8 Graduate Institute of Clinical Medicine, College of Medicine, Kaohsiung Medical University, Kaohsiung 807378, Taiwan.; 9 Department of Urology, School of Medicine, College of Medicine, Kaohsiung Medical University 807378, Kaohsiung, Taiwan.

**Keywords:** kidney stone disease, body mass index, Mendelian randomization, single nucleotide polymorphisms, genetic instrumental variables, obesity, Taiwanese population

## Abstract

**Introduction:**

Kidney stone disease (KSD) is a common urological disorder with an increasing incidence worldwide. Previous observational studies have reported an association between body mass index (BMI) and KSD; however, the causal relationship remains uncertain, particularly in Asian populations. This study aimed to investigate the causal effect of BMI on KSD risk in Taiwanese individuals using a Mendelian randomization (MR) approach.

**Materials and Methods:**

BMI-associated single nucleotide polymorphisms (SNPs) were identified through a literature review and validated in Taiwanese cohorts (n = 107,191). Significant SNPs (*P*-value < 0.05) were selected as instrumental variables for MR analysis, with KSD as the outcome. Several MR methods, including inverse variance weighted (IVW), median-based, robust, and MR-Egger approaches, were applied to estimate the causal effect.

**Results:**

A total of 17 BMI-associated SNPs validated in the Taiwanese cohort were used as instrumental variables in the MR analysis. The penalized IVW model demonstrated a significant positive association between genetically predicted BMI and KSD risk (OR = 2.16, 95% CI: 1.22-3.81, *P*-value = 0.008). Similar results were observed using robust IVW (OR = 2.13, 95% CI: 1.25-3.62, *P*-value = 0.005) and weighted median approaches (*P*-value = 0.050 and 0.042). No evidence of directional pleiotropy was detected.

**Conclusion:**

Our findings provide genetic evidence supporting a causal association between higher BMI and increased KSD risk in Taiwanese individuals, suggesting that weight management may play an important role in KSD prevention. Further studies are needed to investigate the underlying biological mechanisms, population-specific genetic susceptibilities, and effective preventive strategies for obesity-related KSD.

## Introduction

Kidney stone disease (KSD) is a common and increasingly prevalent urological condition that imposes a significant healthcare burden globally. 1 It is characterized by the formation of calculi within the urinary tract, often leading to acute pain, hematuria, urinary tract obstruction, and recurrent hospitalizations. 2, 3 The global prevalence of KSD is estimated to range from 5% to 15%, with notable regional and ethnic differences. 1, 4 In Taiwan, the incidence of KSD has shown a steady rise over recent decades, possibly driven by changes in diet, lifestyle, and comorbidity profiles. 5 Identifying modifiable risk factors is essential to developing effective preventive strategies and reducing the disease burden.

Obesity, often measured by body mass index (BMI), has emerged as a potential risk factor for KSD in multiple observational studies. 6-8 Obese individuals have been shown to have a higher risk of developing kidney stones due to alterations in urinary composition, including increased excretion of calcium, oxalate, uric acid, and other lithogenic factors. 7 However, most evidence linking BMI and KSD has been derived from observational studies, which are susceptible to residual confounding, reverse causation, and measurement bias. 9 The true causal relationship between BMI and KSD, especially in Asian populations, remains unclear. This is of particular importance since Asian individuals, including Taiwanese, may differ in genetic backgrounds, body composition, and stone composition compared to Western populations. 10, 11

Mendelian Randomization (MR) offers a powerful approach to overcoming the limitations of observational research by leveraging genetic variants as instrumental variables (IVs) to infer causality. 12 Since genetic variants are randomly assigned at conception and generally not affected by confounders, MR mimics the design of a natural randomized controlled trial. 13, 14 Prior MR studies in Western populations have suggested a possible causal role of higher BMI in increasing the risk of KSD. 15 However, the generalizability of these findings to East Asian populations, such as Taiwanese, is uncertain due to ethnic-specific genetic and environmental differences. Notably, few, if any, MR studies have directly addressed the BMI-KSD relationship in Taiwanese individuals.

In this study, we aimed to investigate the causal relationship between BMI and KSD in a Taiwanese population using MR approach, including inverse variance weighted (IVW), penalized and robust models, as well as MR-Egger regression, were applied to ensure robustness and sensitivity of the causal estimates. By doing so, we sought to provide more definitive evidence on whether BMI plays a causal role in the risk of KSD in Taiwanese individuals, which may inform public health strategies and clinical management focused on weight control for KSD prevention.

## Materials and Methods

### Study Population and Cohort Description

**Figure 1:**
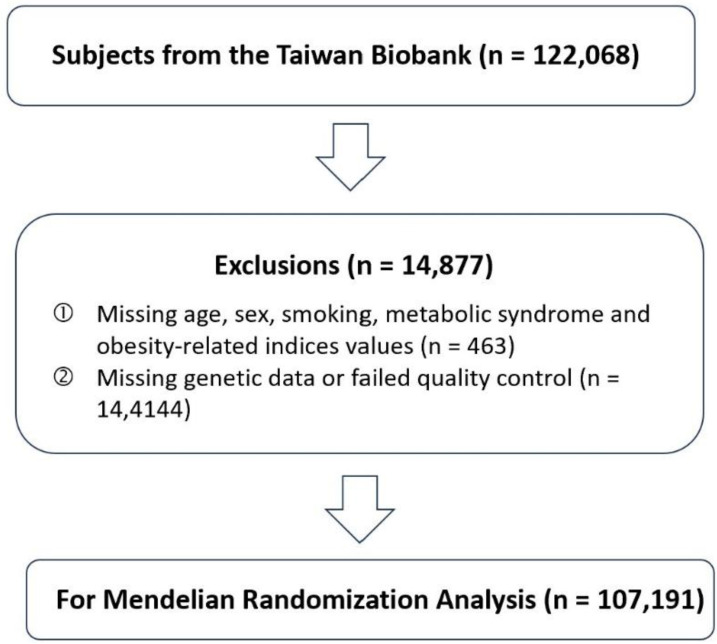
Flowchart of Participant Selection for Mendelian Randomization Analysis. This flowchart illustrates the selection process of study participants from the Taiwan Biobank. A total of 122,068 individuals were initially enrolled. After excluding participants with missing demographic, lifestyle, or metabolic information (n = 463) and those with missing or low-quality genetic data (n = 14,414), 107,191 participants remained eligible and were included in the Mendelian randomization (MR) analysis.

A total of 122,068 participants from the Taiwan Biobank (TWB) were initially considered for inclusion and detailed information about the TWB has been described in previous publications. 16-21 Participants with missing information on key variables, including age, sex, smoking status, metabolic syndrome, or obesity-related indices, were excluded (n = 463). In addition, individuals with incomplete or low-quality genetic data were removed from the analysis (n = 14,414). After these exclusions, 107,191 participants remained and were included in the MR analysis (**Figure 1**). Detailed phenotypic information, including height, weight, and KSD diagnosis, was collected through structured interviews, clinical examination, and linked health records. BMI was calculated as weight in kilograms divided by height in meters squared (kg/m²). The diagnosis of KSD was based on self-report diagnosed KSD. All participants provided written informed consent, and the study protocol was approved by the Institutional Review Board of the affiliated research institutions (KMUHIRB-E(I)-20210058).

### Genotyping and Quality Control

Genotyping data were obtained using the customized Axiom TWB 2.0 array platform. 18 Rigorous quality control (QC) measures were implemented to ensure data reliability. Single nucleotide polymorphisms (SNPs) were filtered out if they exhibited a call rate below 98%, a minor allele frequency (MAF) less than 1%, or significant deviation from Hardy-Weinberg equilibrium (*P*-value < 1×10⁻⁶). In addition, individuals showing unexpected relatedness, discrepancies between reported and genetic sex, or genotype call rates lower than 95% were excluded from further analyses.

### Selection and Validation of Instrumental Variables

We extracted SNPs significantly associated with BMI from the GWAS catalog 22 and further validated their effects in our Taiwanese cohort. Thirty-six independent SNPs were selected based on linkage disequilibrium pruning (r² < 0.01) and their biological relevance to BMI. Variants that met the genome-wide significance threshold (*P-*value < 1×10⁻⁸) and demonstrated consistent effect directions in our population were retained as instrumental variables. Details of each SNP, including effect allele, beta coefficient, standard error, and minor allele frequency, are presented in **Supplementary Tables 1 and 2**.

Instrument strength was assessed using the F-statistic calculated as 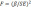, where β and SE represent the SNP-BMI association estimates in the Taiwanese cohort. Across the 17 validated SNPs included in the primary analysis, the mean F-statistic was 12.29 (range 4.05-38.02) (**Supplementary Table 3**).

### Observational Association Between BMI and KSD

To evaluate the observational association between BMI and KSD, univariate and multivariate logistic regression models were constructed. Odds ratios (ORs) with 95% confidence intervals (CIs) were estimated. The multivariate model adjusted for age, sex, smoking status, and metabolic syndrome. BMI was modeled as a continuous variable.

### Mendelian Randomization Analyses

MR analyses were performed using multiple complementary statistical approaches to estimate the causal effect of genetically predicted BMI on the risk of KSD. The IVW method served as the primary analysis under the assumption that all instruments were valid. To assess the robustness of the findings and account for potential pleiotropy, several additional estimators were applied, including the simple median, weighted median, and penalized weighted median methods, the latter two providing reliable estimates even when up to 50% of the weight comes from invalid instruments. MR-Egger regression was used to evaluate directional pleiotropy through its intercept term, while robust IVW and penalized robust IVW methods were applied to mitigate the influence of outlier variants. Causal estimates were expressed as ORs with corresponding 95% CIs and *P*-values. Heterogeneity and directional pleiotropy were assessed using the MR-Egger intercept test. All analyses were conducted in R (version 4.5.2; R Foundation for Statistical Computing, Vienna, Austria) using the *MendelianRandomization* package (version 0.10.0). 23 Because genetic variants are randomly allocated at conception, MR analyses were performed without adjustment for conventional confounders such as age, sex, or smoking status.

### Sensitivity Analyses

Sensitivity analyses were conducted to examine the robustness of the MR results. The MR-PRESSO (Pleiotropy Residual Sum and Outlier) test was used to detect horizontal pleiotropy and identify potential outlier SNPs. Both the raw and outlier-corrected MR-PRESSO estimates were obtained, and the global test statistic was used to evaluate the overall presence of pleiotropy. To further account for the potential impact of multiple testing during SNP validation, an additional sensitivity analysis was performed using a Bonferroni-corrected threshold. Specifically, SNPs that did not meet the significance criterion of *P-*value < 0.0029 in the Taiwanese cohort were excluded, and the MR analyses were repeated using only the remaining variants.

### Statistical Analyses

Continuous variables were presented as mean values with corresponding standard deviations, while categorical variables were expressed as frequencies and percentages. Differences between participants with and without KSD were assessed using independent *t*-tests for continuous variables and chi-square tests for categorical variables. MR analyses were conducted in R (version 4.5.2; R Foundation for Statistical Computing, Vienna, Austria) and Rstudio (version 2025.09.0; Posit Software, PBC, Boston, MA, USA), using customized scripts for sensitivity testing and assessment of instrument strength.

## Results

### Clinical Characteristics of the Study Population

Our study population included 107,191 participants, with a mean age of 49.9 years and a mean BMI of 24.2 kg/m². Among them, 68,620 were female and 38,571 were male (**Table 1**). In addition, 27.2% had a history of smoking, and 22.5% met the criteria for metabolic syndrome. When separating participants with and without KSD, 6,868 and 100,323 individuals were identified, respectively. Participants with KSD were older (mean age 53.7 vs. 49.6 years) and had higher BMI (25.3 vs. 24.1 kg/m²), a greater prevalence of smoking history (41.5% vs. 26.3%) and metabolic syndrome (33.3% vs. 21.8%), and a higher proportion of males (60.1% vs. 34.3%) compared with those without KSD (**Table 1**).

### Association Between BMI and KSD

To further examine the association between BMI and KSD, we performed a multivariate logistic regression analysis adjusted for sex, age, smoking history, and the presence of metabolic syndrome (**Table 2**). BMI was significantly associated with higher odds of having KSD (Adjusted OR = 1.03, 95% CI = 1.02-1.04, *P*-value < 0.001), indicating that individuals with higher BMI were more likely to have KSD.

### Validation of BMI-Associated Genetic Variants in the Taiwanese Cohort

Among the 36 BMI-associated SNPs identified from previous genome-wide association studies (**Supplementary Tables 1 and 2**) 22, 17 passed quality control and were validated at the significance threshold (*P*-value < 0.05) in the Taiwanese cohort (**Table 3**). These validated variants were distributed across multiple chromosomes and exhibited consistent effect directions with previously reported BMI associations. Several SNPs demonstrated particularly strong associations with BMI in the Taiwanese population, including rs4077410 (*TAOK2*, Chr 16, β = 0.1011, *P*-value = 6.89 × 10⁻¹⁰, MAF = 0.4392), rs591120 (*SEC16B*, Chr 1, β = 0.0818, *P*-value = 4.82 × 10⁻⁶, MAF = 0.2986), rs9891146 (*C17orf58*, Chr 17, β = -0.0955, *P*-value = 1.89 × 10⁻⁷, MAF = 0.2851), and rs1064608 (*MTCH2*, Chr 11, β = 0.0700, *P*-value = 9.67 × 10⁻⁵, MAF = 0.2928 (**Table 3 and Supplementary Table 4**). The direction and magnitude of these effects were comparable to findings in previous GWAS, supporting the robustness of these genetic instruments. Using these validated SNPs as instrumental variables, subsequent MR analyses were conducted to estimate the causal effect of genetically predicted BMI on the risk of KSD.

### Causal Effect of Genetically Predicted BMI on KSD

**Figure 2:**
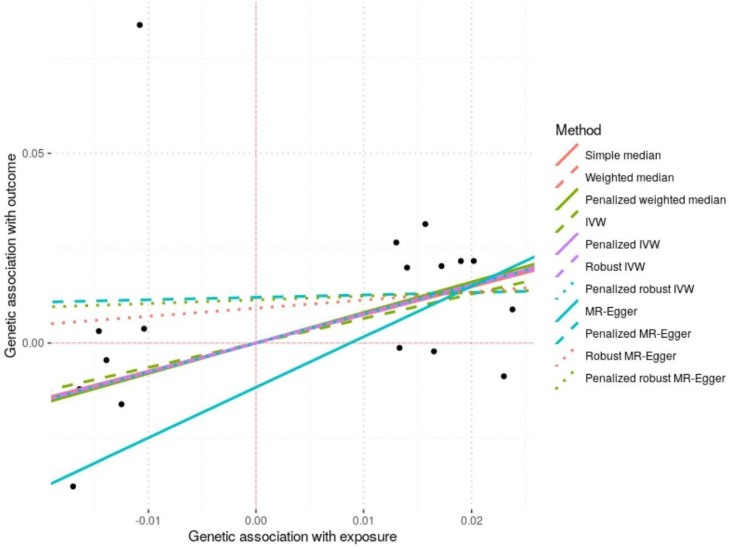
Scatter Plot of Mendelian Randomization Analyses for the Effect of Genetically Predicted BMI on KSD. Each black point represents the genetic association of an individual BMI-associated SNP with kidney stone disease (KSD) on the y-axis versus its association with body mass index (BMI) on the x-axis. The fitted lines correspond to causal estimates obtained from different Mendelian randomization (MR) methods, including simple median, weighted median, penalized weighted median, inverse-variance weighted (IVW), penalized IVW, robust IVW, penalized robust IVW, MR-Egger, penalized MR-Egger, robust MR-Egger, and penalized robust MR-Egger. A positive slope indicates a positive causal effect of genetically predicted BMI on the risk of KSD. The overall consistency across methods supports the reliability of the causal association.

MR analyses were conducted using the 17 BMI-associated SNPs validated in the Taiwanese cohort to assess the causal effect of genetically predicted BMI on KSD (**Table 4A** and **4B**, **Figure 2**). The IVW method suggested a positive causal effect (OR = 1.91, 95% CI = 0.99-3.67, *P*-value = 0.054), which reached statistical significance in the penalized IVW (OR = 2.16, 95% CI = 1.22-3.81, *P*-value = 0.008) and robust IVW analyses (OR = 2.13, 95% CI = 1.25-3.62, *P*-value = 0.005). Consistent findings were observed with the weighted median and penalized weighted median methods (*P*-value = 0.050 and 0.042, respectively), supporting the robustness of the association. MR-Egger regression yielded a directionally similar but imprecise estimate (OR = 3.78, 95% CI = 0.18-78.5, *P*-value = 0.389). No evidence of directional horizontal pleiotropy was detected based on MR-Egger intercept tests (all *P*-value > 0.05).

When restricting the analysis to SNPs meeting the Bonferroni-corrected threshold (*P*-value < 0.0029), eight variants remained as instrumental variables (**Supplementary Table 5**). MR analyses using this reduced set of SNPs yielded effect estimates that were directionally consistent with the primary analysis but were no longer statistically significant, reflecting the reduced statistical power associated with fewer instruments (**Supplementary Tables 5-6 and Supplementary Figure 1**).

### Sensitivity Analysis and Assessment of Horizontal Pleiotropy

Sensitivity analyses using MR-PRESSO detected evidence of horizontal pleiotropy (global test: RSSobs = 2015.87, *P*-value < 0.001); however, after outlier correction, the causal estimate remained directionally consistent and nonsignificant changes were observed (raw estimate = 0.17, *P*-value = 0.25; outlier-corrected estimate = 0.49, *P*-value = 0.27), suggesting that no single variant disproportionately influenced the overall result (**Table 5**). MR-Egger intercept tests also indicated no evidence of directional pleiotropy (*P*-value > 0.05) (**Table 4B**). Although MR-PRESSO suggested the presence of heterogeneity among SNPs, this does not necessarily imply directional pleiotropy but may reflect variability in SNP-specific causal estimates.

## Discussion

This study results provide robust genetic evidence, which supports a positive association between genetically predicted BMI and risk of KSD in a Taiwanese population. These findings underscore the importance of weight management as a preventive strategy against KSD, particularly within East Asian populations where evidence on this relationship has previously been limited.

Several observational studies have consistently reported associations between obesity or increased BMI and KSD risk. 6-8 A transnational meta-analysis including 8 cohort studies found that per 5 kg/m2 increase in BMI increased the risk of kidney stones formation by 21%, and this association remained robust after removing of heterogenous studies. 24 Another cohort study with about 270,000 North-East Asian population, showed obesity were associated with an increased risk of kidney stones after a median of 4-year follow-up period. 25 Despite these observational correlations, residual confounding and reverse causation issues have made causal inference challenging. 9

In the present study, MR was used to better evaluate whether the association between BMI and KSD may reflect a causal relationship. Because genetic variants are randomly assigned at conception according to Mendel's laws of inheritance 14, they are less likely to be influenced by lifestyle or environmental confounding factors. Using BMI-associated genetic variants as instruments, we observed consistent results across IVW, robust IVW, and median-based methods, suggesting that higher genetically predicted BMI is associated with an increased risk of KSD. Although MR-Egger regression was not statistically significant, no evidence of directional pleiotropy was detected. Overall, these findings support a likely causal relationship between higher BMI and KSD risk in this Taiwanese population.

Our findings are consistent with prior MR studies conducted primarily in Western populations. Shuai Yuan et al demonstrated a high BMI and type 2 diabetes were causally associated with an elevated risk of kidney stones in two independent population. 15 Moreover, the magnitude of this causal relationship observed in our study highlights potential ethnic differences in genetic susceptibility of BMI-related risk. Such differences could reflect variations in genetic architecture, dietary patterns, metabolic profiles, or urinary compositions specific to Asian populations. Further genetic and epidemiological studies across diverse ethnicities are warranted to clarify these population-specific relationships and identify underlying mechanisms.

Although the present study did not directly examine biological pathways, several plausible mechanisms have been proposed in previous studies that may help explain the relationship between obesity and KSD. 1 Higher BMI is known to alter urinary biochemical profiles, not only promoting stone formation through increased excretion of lithogenic substances such as calcium, oxalate, and uric acid, but also decrease concentration of anti-lithogenic substance such as citrate in urine. Obesity-related insulin resistance and metabolic syndrome contribute to altered renal handling of electrolytes and urinary acidification, resulting in a lower urine pH level and lithogenic urinary environment. 7, 26 Furthermore, chronic inflammation and oxidative stress that are closely associated with obesity related adiposity also play a role in kidney stones formation. 27, 28 Moreover, the obesity-related hormonal changes, particularly in adipokines such as leptin and adiponectin, could modulate renal tubular function and stone-forming processes. Leptin, often elevated in obese individuals, has been suggested to influence renal calcium transport and excretion negatively. 29 Conversely, adiponectin levels, typically reduced in obesity, play protective roles against insulin resistance and inflammation, both critical in preventing stone formation. 30 Taken together, these mechanisms may partially explain the observed relationship between BMI and KSD. However, these mechanisms remain hypothesis-generating and require further experimental and clinical studies to confirm.

The robust methodology employed in this study, particularly the rigorous selection and validation of instrumental SNPs, strengthens the validity of results. Nevertheless, some limitations warrant consideration. Firstly, our analyses were limited to common SNPs identified through GWAS, potentially overlooking rarer genetic variants with larger effects. Future genetic studies using whole-exome or whole-genome sequencing may reveal additional instrumental variables with stronger or more direct biological relevance. Secondly, the MR approach relies on several critical assumptions, primarily the absence of horizontal pleiotropy, where genetic variants influence KSD risk through pathways other than BMI. Although our sensitivity analyses and MR-Egger intercept tests suggested minimal pleiotropy, the possibility of residual pleiotropic effects cannot be entirely dismissed. Future studies integrating omics data could provide deeper insights into the pleiotropic mechanisms and help refine causal estimates. Thirdly, both the SNP-BMI and SNP-KSD associations were estimated within the same TWB dataset, resulting in a one-sample MR design. In contrast to two-sample MR, weak instrument bias in one-sample MR may bias causal estimates toward the corresponding observational association. Although the mean F-statistic indicated generally acceptable instrument strength, residual bias cannot be entirely excluded. Therefore, the magnitude of the causal estimates should be interpreted cautiously. Fourthly, KSD was defined based on self-reported physician-diagnosed history in the Taiwan Biobank, which may introduce outcome misclassification. However, such misclassification is likely to be non-differential with respect to genetic variants and would tend to bias MR estimates toward the null rather than generate spurious associations. Fifthly, because the genetic instruments, exposure distributions, and outcome ascertainment were derived from the TWB, the findings should be interpreted primarily within the Taiwanese population. Replication in other ethnic and geographic populations will be necessary to determine the generalizability of these results. Finally, BMI as a single measure of adiposity might not fully capture the complexity of obesity, particularly body fat distribution and metabolic health. Metrics such as waist circumference, visceral fat measurements, and metabolic parameters might provide more detailed insights into obesity-related KSD risks in future studies.

Clinically, our study emphasizes BMI control as a potentially effective intervention strategy for reducing KSD risk. Given the rising trends in obesity and KSD prevalence globally, integrating weight management programs with standard urological care could substantially impact public health outcomes. Future research could explore personalized intervention strategies targeting genetically susceptible individuals, aiming for more precise prevention of KSD through weight management and lifestyle modifications.

## Conclusion

In conclusion, our MR study provides genetic evidence suggesting that higher BMI may causally increase the risk of KSD in Taiwanese individuals. The consistency of the findings across multiple MR methods supports the robustness of the observed association. These results suggest that weight management may play an important role in KSD prevention. Further research exploring underlying biological mechanisms, ethnic-specific genetic susceptibility, and preventive strategies is warranted to better understand and mitigate the burden of KSD associated with obesity.

## Supplementary Material

Supplementary figures and tables.

## Figures and Tables

**Table 1:** Baseline characteristics of the study population.

Variable	Overall (N = 107,191)	Non-KSD (N = 100,323)	KSD (N = 6,868)	*P*-value
Sex, n (%)				< 0.001
Male	38,571 (36.0)	34,443 (34.3)	4,128 (60.1)	
Female	68,620 (64.0)	65,880 (65.7)	2,740 (39.9)	
Age, mean (SD), years	49.9 (10.9)	49.6 (11.0)	53.7 (9.7)	< 0.001
Smoking experience, n (%)				< 0.001
No	78,004 (72.8)	73,988 (73.7)	4,016 (58.5)	
Yes	29,187 (27.2)	26,335 (26.3)	2,852 (41.5)	
BMI, mean (SD), kg/m²	24.2 (3.8)	24.1 (3.8)	25.3 (3.8)	< 0.001
Metabolic syndrome, n (%)				< 0.001
No	83,028 (77.5)	78,449 (78.2)	4,579 (66.7)	
Yes	24,163 (22.5)	21,874 (21.8)	2,289 (33.3)	

Abbreviations: KSD = Kidney stone disease; BMI = Body mass index; SD = Standard deviation.

**Table 2:** Multivariate logistic regression analysis of risk factors associated with kidney stone disease.

Variables	Adjusted OR	95% CI	*P*-value
Female sex (vs. male)	0.43	0.40-0.46	< 0.001
Age (per 1-year increase)	1.03	1.03-1.03	< 0.001
Smoking (yes vs. no)	1.13	1.07-1.20	< 0.001
BMI (per 1 kg/m² increase)	1.03	1.02-1.04	< 0.001
Metabolic syndrome (yes vs. no)	1.15	1.08-1.22	< 0.001

Abbreviations: OR = odds ratio; CI = confidence interval; BMI = body mass index. Note: Adjusted for sex, age, smoking status, BMI, and metabolic syndrome.

**Table 3:** Validation of BMI-Associated SNPs in the Taiwanese Cohort showing significant *P*-values

SNP ID	Chr	Position (hg38)	Mapped Gene(s)	Effect Allele	Other Allele	MAF	β (BMI)	SE	*P*-value
rs2297792	1	156041653	*UBQLN4*	C	T	0.1157	0.1025	0.0254	5.58×10⁻⁵
rs591120	1	177933618	*SEC16B*	C	G	0.2986	0.0818	0.0179	4.82×10⁻⁶
rs2230590	3	49898669	*MST1R*	C	T	0.1413	0.0821	0.0235	4.85×10⁻⁴
rs1052618	3	136855659	*SLC35G2*	A	G	0.1322	-0.0504	0.0241	0.0362
rs9438	3	154301098	*DHX36*	C	G	0.4499	0.0330	0.0164	0.0440
rs11755393	6	34856859	*BLTP3A*	G	A	0.4907	0.0430	0.0163	0.0084
rs10829163	10	27028911	*ANKRD26*	C	T	0.4388	-0.0385	0.0164	0.0190
rs284860	10	102813206	*WBP1L*	C	T	0.3987	-0.0439	0.0168	0.0089
rs11042023	11	8640969	*TRIM66*	C	T	0.4086	0.0386	0.0166	0.0202
rs11555762	11	43855148	*HSD17B12*	T	C	0.2628	0.0453	0.0186	0.0149
rs1064608	11	47618877	*MTCH2*	C	G	0.2928	0.0700	0.0180	9.67×10⁻⁵
rs12828016	12	889199	*WNK1*	T	G	0.2758	-0.0400	0.0182	0.0281
rs1131877	14	102875712	*TRAF3*	C	T	0.3958	0.0530	0.0167	0.0015
rs11071896	15	66528912	*ZWILCH*	G	A	0.1706	-0.0451	0.0217	0.0376
rs4077410	16	29986879	*TAOK2*	G	A	0.4392	0.1011	0.0164	6.89×10⁻¹⁰
rs2306590	17	36498436	*MYO19*	A	G	0.3097	-0.0694	0.0177	8.79×10⁻⁵
rs9891146	17	67991933	*C17orf58*	C	T	0.2851	-0.0955	0.0183	1.89×10⁻⁷

β represents the per-allele effect size on BMI (kg/m²) in the Taiwanese validation cohort. MAF denotes minor allele frequency. Detailed genomic context and functional annotations are provided in Supplementary Tables 1-3. Abbreviations: SNP = single nucleotide polymorphism; Chr = chromosome; MAF = minor allele frequency; SE = standard error; BMI = body mass index.

**Table 4A:** Causal Effect Estimates. Mendelian Randomization Estimates for the Effect of Genetically Predicted BMI on KSD

Method	OR	95% CI	*P*-value
IVW	1.91	0.99-3.67	0.054
Penalized IVW	2.16	1.22-3.81	0.008
Robust IVW	2.13	1.25-3.62	0.005
Penalized robust IVW	2.15	1.22-3.80	0.008
Weighted median	2.17	1.00-4.72	0.050
Penalized weighted median	2.24	1.03-4.86	0.042
Simple median	2.10	0.92-4.76	0.076
MR-Egger (slope)	3.78	0.18-78.5	0.389

**Table 4B:** Assessment of Horizontal Pleiotropy. Mendelian Randomization Estimates for the Effect of Genetically Predicted BMI on KSD

Method	Intercept (β)	SE	*P*-value
MR-Egger	-0.0117	0.0256	0.649
Penalized MR-Egger	0.0120	0.0226	0.595
Robust MR-Egger	0.0092	0.0182	0.614
Penalized robust MR-Egger	0.0114	0.0189	0.548

Effect estimates are presented as odds ratios (ORs) with 95% confidence intervals (CIs) for kidney stone disease per 1-unit increase in genetically predicted BMI, consistent with the scale of the SNP-BMI association used in the instrumental variable analysis. MR models were not adjusted for demographic or clinical covariates, as genetic instrumental variables are assumed to be randomly allocated at conception and therefore independent of these potential confounders. Abbreviations: BMI = body mass index; MR = Mendelian randomization; OR = odds ratio; CI = confidence interval; SE = standard error; IVW = inverse-variance weighted; MR-Egger = Mendelian randomization Egger regression.

**Table 5:** Sensitivity Analyses for Pleiotropy and Outlier Correction

Analysis	Causal Estimate	SD	T-stat	*P*-value
Raw MR-PRESSO estimate	0.169	0.143	1.184	0.254
Outlier-corrected MR-PRESSO estimate	0.486	0.360	1.349	0.270
Test		Statistic		*P*-value
MR-PRESSO global test (RSSobs)		2015.87		<0.001

**Abbreviations:** MR-PRESSO = Mendelian randomization pleiotropy residual sum and outlier; SD = standard deviation; RSSobs = observed residual sum of squares.

## Data Availability

The data underlying this study are from the Taiwan Biobank. Due to restrictions placed on the data by the Personal Information Protection Act of Taiwan, the minimal data set cannot be made publicly available. Data may be available upon request to interested researchers. Please send data requests to: Jiun-Hung Geng, MD. Department of Urology, Kaohsiung Municipal Siaogang Hospital.
