# Supplementary material for: Causal Association Between Body Mass Index and Kidney Stone Disease in Taiwanese: A Mendelian Randomization Study
Source: Int J Med Sci. 2026 Mar 17;23(5):1605–12. doi: 10.7150/ijms.128104 (PMC13133876; doi:10.7150/ijms.128104)
Supplement: Supplementary file 1 — Supplementary figures and tables. [file ijmsv23p1605s1.pdf]

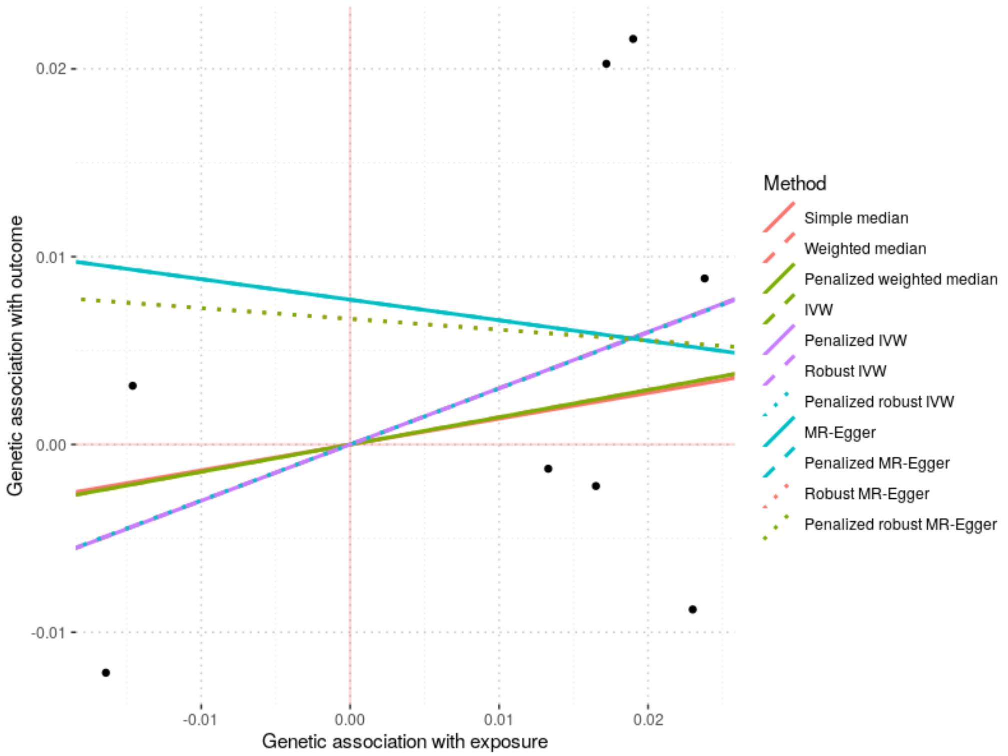

Supplementary Figure 1. Scatter plot of Mendelian randomization analysis using Bonferroni-selected SNPs. Scatter plot showing the association between genetic variants and body mass index (BMI) on the x-axis and kidney stone disease (KSD) on the y-axis for the eight SNPs retained after Bonferroni correction. Each point represents an individual SNP used as an instrumental variable. The fitted lines correspond to causal estimates derived from different Mendelian randomization methods, including inverse-variance weighted (IVW), weighted median, and MR-Egger regression. Although the estimates were not statistically significant due to the reduced number of instruments, the overall direction of effect remained consistent with the primary analysis, suggesting a positive relationship between genetically predicted BMI and KSD risk.

Supplementary Table 1. Summary of BMI-Associated SNPs Identified From Previous Genome-Wide Association Studies (*Nat Genet*. 2018;50(1):26-41.)

SNP ID	Chromosome	Position	Reported Gene(s)	Mapped Gene	Context	Risk Allele	Risk Allele Frequency	β (Effect)	SE	*P*-value
rs1052618	3	136855659	SLC35G2	NCK1-DT, SLC35G2	Missense	G	0.693	0.0108	0.0019	8.0×10^{-9}
rs1064608	11	47618877	MTCH2	MTCH2	Missense	C	0.3365	0.0238	0.0020	1.0×10^{-31}
rs1071648	17	5422769	RPAIN	RPAIN	Missense	T	0.2874	0.0124	0.0020	5.0×10^{-10}
rs10829163	10	27028911	ANKRD26	ANKRD26	Missense	T	0.1616	0.0170	0.0026	2.0×10^{-12}
rs11042023	11	8640969	TRIM66	TRIM66	Missense	C	0.6395	0.0157	0.0018	1.0×10^{-16}
rs11071896	15	66528912	ZWILCH	ZWILCH	Missense	A	0.7501	0.0125	0.0020	6.0×10^{-10}
rs1131877	14	102875712	TRAF3, XRCC3	TRAF3	Missense	C	0.2518	0.0172	0.0020	2.0×10^{-16}
rs11555762	11	43855148	HSD17B12	HSD17B12	Missense	T	0.3104	0.0140	0.0020	5.0×10^{-14}
rs1169081	12	121968006	WDR66, MLXIP	CFAP251	Missense	G	0.7040	0.0122	0.0019	3.0×10^{-10}
rs11755393	6	34856859	UHRF1BP1, PACSIN1	BLTP3A	Missense	G	0.3556	0.0202	0.0020	3.0×10^{-26}
rs12828016	12	889199	WNK1	WNK1	Missense	G	0.6132	0.0139	0.0018	5.0×10^{-15}

SNP ID	Chromosome	Position	Reported Gene(s)	Mapped Gene	Context	Risk Allele	Risk Allele Frequency	β (Effect)	SE	*P*-value
rs1539172	9	15784633	CCDC171	CCDC171	Missense	G	0.4956	0.0111	0.0017	1.0×10^{-10}
rs1801265	1	97883329	DPYD	DPYD	Missense	G	0.2334	0.0127	0.0021	9.0×10^{-10}
rs2075803	19	51125272	SIGLEC9	SIGLEC9	Stop gained	G	0.5583	0.0099	0.0017	1.0×10^{-8}
rs2076559	20	25206577	ENTPD6	ENTPD6	Missense	A	0.3279	0.0155	0.0018	2.0×10^{-16}
rs2228273	20	18315432	ZNF133	ZNF133	Missense	A	0.0819	0.0238	0.0031	6.0×10^{-14}
rs2230590	3	49898669	MST1R, MON1A, HYAL3	MST1R	Missense	C	0.4916	0.0190	0.0020	9.0×10^{-26}
rs2277598	15	72735137	BBS4	BBS4	Missense	C	0.6277	0.0148	0.0020	2.0×10^{-15}
rs2280843	9	128822790	C9orf114	SPOUT1, KYAT1	Missense	G	0.7310	0.0146	0.0027	1.0×10^{-7}
rs2306590	17	36498436	MYO19	MYO19, ZNHIT3	Missense	G	0.6099	0.0164	0.0018	2.0×10^{-18}
rs2396359	19	1819126	REXO1	REXO1	Missense	T	0.7638	0.0147	0.0023	2.0×10^{-11}
rs284860	10	102813206	WBP1L	WBP1L	Missense	T	0.4112	0.0104	0.0018	9.0×10^{-9}
rs3088142	10	75094806	DUSP13	DUSP13	Missense	T	0.4332	0.0173	0.0033	5.0×10^{-8}
rs3184504	12	111446804	SH2B3	SH2B3, ATXN2	Missense	C	0.5548	0.0129	0.0019	7.0×10^{-12}

SNP ID	Chromosome	Position	Reported Gene(s)	Mapped Gene	Context	Risk Allele	Risk Allele Frequency	β (Effect)	SE	*P*-value
rs3213758	16	53605526	RPGRIP1L	RPGRIP1L	Missense	C	0.9432	0.0247	0.0038	5.0×10^{-11}
rs3760128	17	75890807	TRIM65	TRIM65	Missense	G	0.3467	0.0126	0.0021	1.0×10^{-9}
rs4077410	16	29986879	TAOK2	TAOK2	Synonymous	G	0.5114	0.0165	0.0018	2.0×10^{-21}
rs459552	5	112841059	APC	APC	Missense	T	0.2220	0.0124	0.0021	5.0×10^{-9}
rs4851287	2	100299310	LONRF2	LONRF2	Missense	A	0.3461	0.0128	0.0018	2.0×10^{-12}
rs56384862	3	58410136	PXK	PXK	Missense	G	0.3382	0.0128	0.0019	8.0×10^{-12}
rs5758651	22	42213142	TCF20	TCF20	Missense	T	0.8029	0.0122	0.0022	3.0×10^{-8}
rs591120	1	177933618	SEC16B	SEC16B	Missense	C	0.4440	0.0230	0.0020	5.0×10^{-38}
rs62623713	1	109476817	SYPL2	SYPL2	Missense	G	0.0600	0.0276	0.0036	4.0×10^{-14}
rs9438	3	154301098	DHX36	DHX36	Missense	C	0.3991	0.0130	0.0018	2.0×10^{-13}
rs9891146	17	67991933	C17orf58	C17orf58	Missense	T	0.2930	0.0146	0.0023	1.0×10^{-11}

Abbreviations: SNP = single nucleotide polymorphism; SE = standard error; BMI = body mass index.

Note: Effect estimates (β) and P-values are reported from Locke et al., Nature (2015) (PubMed ID: 29273807).

Supplementary Table 2. Validation of BMI-Associated SNPs in the Taiwanese Cohort

SNP ID	β	SE	*P*-value	Reference Allele	Alternate Allele	Effect Allele	SNP ID
rs1801265	-0.0394	0.0315	0.2119	A	G	G	A
rs62623713	-0.0052	0.1848	0.9777	A	G	G	A
rs2297792	0.1025	0.0254	5.58×10^{-5}	T	C	C	T
rs591120	0.0818	0.0179	4.82×10^{-6}	G	C	C	G
rs4851287	0.0383	0.0379	0.3126	G	A	A	G
rs2230590	0.0821	0.0235	4.85×10^{-4}	T	C	C	T
rs56384862	0.2920	0.2316	0.2073	A	G	G	A
rs1052618	-0.0504	0.0241	0.0362	G	A	A	G
rs9438	0.0330	0.0164	0.0440	G	C	C	G
rs459552	0.0251	0.0271	0.3532	A	T	T	A
rs11755393	0.0430	0.0163	0.0084	A	G	G	A
rs1539172	0.0075	0.0171	0.6626	A	G	G	A
rs2280843	-0.0011	0.0183	0.9501	G	A	A	G
rs10829163	-0.0385	0.0164	0.0190	T	C	C	T
rs3088142	-0.0497	0.0281	0.0763	T	C	C	T
rs284860	-0.0439	0.0168	0.0089	T	C	C	T
rs11042023	0.0386	0.0166	0.0202	T	C	C	T

SNP ID	β	SE	*P*-value	Reference Allele	Alternate Allele	Effect Allele	SNP ID
rs11555762	0.0453	0.0186	0.0149	C	T	T	C
rs1064608	0.0700	0.0180	9.67×10^{-5}	G	C	C	G
rs12828016	-0.0400	0.0182	0.0281	G	T	T	G
rs3184504	-0.0397	0.3322	0.9049	C	T	T	C
rs1169081	0.0204	0.0163	0.2109	T	G	G	T
rs1131877	0.0530	0.0167	0.0015	T	C	C	T
rs11071896	-0.0451	0.0217	0.0376	A	G	G	A
rs2277598	0.0312	0.0190	0.1003	T	C	C	T
rs4077410	0.1011	0.0164	6.89×10^{-10}	A	G	G	A
rs3213758	0.0094	0.0175	0.5918	C	T	T	C
rs1071648	-0.0093	0.0215	0.6671	T	C	C	T
rs2306590	-0.0694	0.0177	8.79×10^{-5}	G	A	A	G
rs9891146	-0.0955	0.0183	1.89×10^{-7}	T	C	C	T
rs3760128	0.0080	0.0204	0.6942	A	G	G	A
rs2396359	-0.0182	0.0165	0.2711	T	C	C	T
rs2075803	0.0055	0.0164	0.7382	G	A	A	G
rs2228273	0.0158	0.0253	0.5322	G	A	A	G
rs2076559	-0.0119	0.0169	0.4818	A	G	G	A

SNP ID	β	SE	*P*-value	Reference Allele	Alternate Allele	Effect Allele	SNP ID
rs5758651	-0.0267	0.0170	0.1170	T	C	C	T

Abbreviations: SNP = single nucleotide polymorphism; SE = standard error; BMI = body mass index.

Note: Effect estimates (β) represent the association between each SNP and BMI in the Taiwanese cohort.

Supplementary Table 3. Supplementary Table 3. Instrument Strength Assessment of BMI-Associated SNPs Used in Mendelian Randomization Analysis

SNP ID	β	SE	F-statistic
rs2297792	0.1025	0.0254	16.28
rs591120	0.0818	0.0179	20.86
rs2230590	0.0821	0.0235	12.18
rs1052618	−0.0504	0.0241	4.37
rs9438	0.0330	0.0164	4.05
rs11755393	0.0430	0.0163	6.95
rs10829163	−0.0385	0.0164	5.50
rs284860	−0.0439	0.0168	6.82
rs11042023	0.0386	0.0166	5.40
rs11555762	0.0453	0.0186	5.93
rs1064608	0.0700	0.0180	15.12
rs12828016	−0.0400	0.0182	4.83
rs1131877	0.0530	0.0167	10.06
rs11071896	−0.0451	0.0217	4.32
rs4077410	0.1011	0.0164	38.02
rs2306590	−0.0694	0.0177	15.36

**SNP ID**	**β**	**SE**	**F-statistic**
rs9891146	−0.0955	0.0183	27.23

Note: The F-statistic was calculated as $F = (\beta/SE)^2$ to assess instrument strength.

Summary statistics (17 SNPs): Mean F = 12.29; Median F = 6.95; Minimum F = 4.05; Maximum F = 38.02.

Abbreviations: SNP = single nucleotide polymorphism; β = regression coefficient representing the SNP–BMI association; SE = standard error; F = F-statistic.

Supplementary Table 4. Functional and Genomic Annotation of BMI-Associated SNPs (*P*-value < 0.05) in the Taiwanese Cohort

SNP	Chr	Region	Position (bp)	Effect Allele	MAF	Mapped Gene(s)
rs1052618	3	3q22.3	136855659	G	0.1322	*NCK1-DT*, *SLC35G2*
rs1064608	11	11p11.2	47618877	C	0.2928	*MTCH2*
rs10829163	10	10p12.1	27028911	T	0.4388	*ANKRD26*
rs11042023	11	11p15.4	8640969	C	0.4086	*TRIM66*
rs11071896	15	15q22.31	66528912	A	0.1706	*ZWILCH*
rs1131877	14	14q32.32	102875712	C	0.3958	*TRAF3*
rs11555762	11	11p11.2	43855148	T	0.2628	*HSD17B12*
rs11755393	6	6p21.31	34856859	G	0.4907	*BLTP3A*
rs12828016	12	12p13.33	889199	G	0.2758	*WNK1*
rs2230590	3	3p21.31	49898669	C	0.1413	*MST1R*
rs2297792	1	1q22	156041653	C	0.1157	*UBQLN4*
rs2306590	17	17q12	36498436	G	0.3097	*MYO19*, *ZNHIT3*
rs284860	10	10q24.32	102813206	T	0.3987	*WBP1L*
rs4077410	16	16p11.2	29986879	G	0.4392	*TAOK2*
rs591120	1	1q25.2	177933618	C	0.2986	*SEC16B*, *CRYZL2P-SEC16B*
rs9438	3	3q25.2	154301098	C	0.4499	*DHX36*
rs9891146	17	17q24.2	67991933	T	0.2851	*C17orf58*

Abbreviations: SNP = single-nucleotide polymorphism; BMI = body mass index; Chr = chromosome; MAF = minor allele frequency.

Supplementary Table 5. BMI-associated SNPs retained after Bonferroni correction for Mendelian randomization sensitivity analysis. This table lists the BMI-associated SNPs that remained significant after applying the Bonferroni-corrected threshold (P-value < 0.05/17 ≈ 0.0029) in the Taiwanese cohort. β represents the per-allele effect size on BMI (kg/m²). Odds ratios (OR) and 95% confidence intervals (CI) were calculated as e^{β} and $e^{(\beta \pm 1.96 \times SE)}$, respectively, to facilitate clinical interpretation. Effect allele indicates the allele associated with increased BMI.

SNP ID	β (BMI)	SE	OR	95% CI	P-value	Effect Allele
rs2297792	0.1025	0.0254	1.108	1.054–1.165	5.58×10^{-5}	C
rs591120	0.0818	0.0179	1.085	1.047–1.124	4.82×10^{-6}	C
rs2230590	0.0821	0.0235	1.085	1.036–1.137	4.85×10^{-4}	C
rs1064608	0.0700	0.0180	1.073	1.036–1.113	9.67×10^{-5}	C
rs1131877	0.0530	0.0167	1.054	1.020–1.089	0.00151	C
rs4077410	0.1011	0.0164	1.106	1.071–1.142	6.89×10^{-10}	G
rs2306590	−0.0694	0.0177	0.933	0.901–0.966	8.79×10^{-5}	A
rs9891146	−0.0955	0.0183	0.909	0.877–0.942	1.89×10^{-7}	C

Abbreviations: SNP = single nucleotide polymorphism; SE = standard error; BMI = body mass index; OR = Odds ratios; CI = confidence intervals.

Supplementary Table 6. Mendelian randomization estimates for the effect of genetically predicted BMI on kidney stone disease using Bonferroni-selected SNPs. Mendelian randomization (MR) analyses were repeated using the eight BMI-associated SNPs that remained significant after Bonferroni correction (P-value < 0.05/17 ≈ 0.0029). Effect estimates are presented as odds ratios (ORs) with 95% confidence intervals (CI) for kidney stone disease per unit increase in genetically predicted BMI. Odds ratios were obtained by exponentiating the regression coefficients from the MR models. IVW = inverse-variance weighted.

**Method**	**OR**	**95% CI**	**P-value**
Simple median	1.15	0.43 – 3.03	0.782
Weighted median	1.16	0.45 – 3.00	0.765
Penalized weighted median	1.16	0.45 – 3.00	0.765
IVW	1.35	0.63 – 2.87	0.436
Penalized IVW	1.35	0.63 – 2.87	0.436
Robust IVW	1.35	0.72 – 2.52	0.353
Penalized robust IVW	1.35	0.72 – 2.52	0.353
MR-Egger	0.90	0.02 – 53.05	0.958

Abbreviations: BMI = body mass index; MR = Mendelian randomization; OR = odds ratio; CI = confidence interval; SE = standard error; IVW = inverse-variance weighted; MR-Egger = Mendelian randomization Egger regression.
